# Age-associated gut microbiota impair hippocampus-dependent memory in a vagus-dependent manner

**DOI:** 10.1172/jci.insight.147700

**Published:** 2022-08-08

**Authors:** Damien Rei, Soham Saha, Marianne Haddad, Anna Haider Rubio, Blanca Liliana Perlaza, Marion Berard, Marie-Noelle Ungeheuer, Harry Sokol, Pierre-Marie Lledo

**Affiliations:** 1 Institut Pasteur de Paris, Paris Cité University, CNRS UMR 3571, Perception and Memory Unit, Paris, France.; 2 Investigation and Access to BioResources (ICAReB) Platform, Paris, France.; 3 Institut Pasteur de Paris, Paris Cité University, DT, Animalerie Centrale, Gnotobiology Center, Paris, France.; 4 Sorbonne University, INSERM, Saint-Antoine Research Center, CRSA, AP-HP, Saint Antoine Hospital, Gastroenterology Department, Paris, France.; 5 INRA, UMR1319 Micalis & AgroParisTech, Jouy en Josas, France.; 6 Paris Center for Microbiome Medicine (PaCeMM) FHU, Paris, France.

**Keywords:** Aging, Neuroscience, Behavior, Mouse models

## Abstract

Aging is known to be associated with hippocampus-dependent memory decline, but the underlying causes of this age-related memory impairment remain highly debated. Here, we show that fecal microbiota transplantation (FMT) from aged, but not young, animal donors into young mice is sufficient to trigger profound hippocampal alterations, including astrogliosis, decreased adult neurogenesis, decreased novelty-induced neuronal activation, and impairment in hippocampus-dependent memory. Furthermore, similar alterations were reported when mice were subjected to an FMT from aged human donors. To decipher the mechanisms involved in mediating these microbiota-induced effects on brain function, we mapped the vagus nerve–related (VN-related) neuronal activity patterns and report that aged FMT animals showed a reduction in neuronal activity in the ascending-VN output brain structure, whether under basal condition or after VN stimulation. Targeted pharmacogenetic manipulation of VN-ascending neurons demonstrated that the decrease in vagal activity is detrimental to hippocampal functions. In contrast, increasing vagal ascending activity alleviated the adverse effects of aged mouse FMT on hippocampal functions and had a promnesic effect in aged mice. Thus, pharmacogenetic VN stimulation is a potential therapeutic strategy to lessen microbiota-dependent age-associated impairments in hippocampal functions.

## Graphical abstract

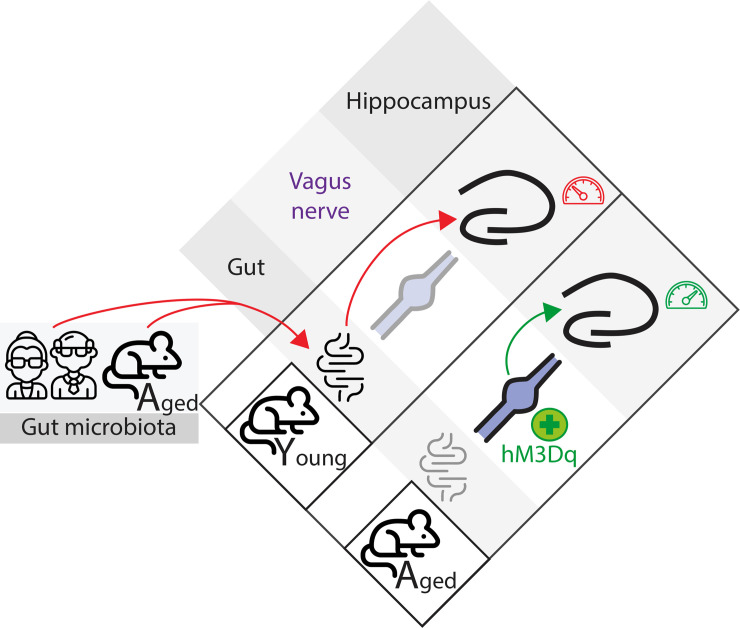

## Introduction

The gut microbiota (GM) — the intestinal community of microorganisms — recently emerged as a key player for physiology and homeostasis, particularly for some brain functions (reviewed in ref. 1), notably learning and memory (2–4). Indeed, the GM influences several hippocampal traits, such as adult neurogenesis (4–6) and astrocyte functions (7), as well as the overall level of brain inflammation (2, 8). Intriguingly, these same brain processes are also affected in aging and lead to the alteration of brain functions and ultimately to hippocampal-dependent impairment in episodic and spatial memory (9). However, understanding the GM’s role on the detrimental effects of the aging process is a daunting task, since age influences the microbiota composition. Therefore, the implication and mechanism of the GM age-associated dysbiosis in the aging phenotype remains mostly unknown.

Evidence is starting to uncover the causal role of GM changes on cognitive functions. In recent studies, GM from aged (A) rodents, transplanted to their young counterparts, impaired learning and memory performances in the Barnes maze (3) or delayed matching to position tests (2). It also promoted systemic (8) and hippocampal inflammation, and it was associated with a perturbation in the expression levels of synaptic plasticity genes (2).

Beyond the possible link via inflammation, the GM is also known to directly communicate with the brain, notably through the vagus nerve (VN). The VN circuit is the most direct and well-studied neuronal pathway of the gut-brain axis (reviewed in ref. 10). VN sensory fibers innervate the muscular and mucosa layers of the gastrointestinal tract, detect mechanosensory and chemical signals, and relay these signals to the CNS through the ventro-medial part of the nucleus tractus solitarius (vmNTS), located in the caudal brainstem (11). Interestingly, activity in this vagal gut-brain circuit is known to modulate hippocampus-related (HPC-related) function. Indeed, the antidepressant effect of probiotic treatment on the hippocampal expression levels of GABA receptors was shown to require an intact VN, since this effect disappears in vagotomized animals (12). Furthermore, VN electrical stimulation is an approved therapy for depression and drug-resistant temporal epilepsy (reviewed in ref. 13), and it is a powerful modulator of hippocampal functions, notably at the electrophysiological (14) and epigenetic (15) levels.

Anatomically, the NTS is connected with the HPC through multisynapse connections — notably through the locus coeruleus and dorsal raphe, which, respectively, release noradrenaline (NA) and serotonin (5-HT) to the HPC and the whole forebrain (16, 17). In line with this, a link between VN ascending neurons and HPC-dependent memory was recently reported using a selective vagal ascending neurons ablation (18) or Ghrelin receptor knock-down (19). Both studies reported an impairment in HPC-dependent episodic and spatial memory. However, beyond rodent GM data, the effect of human age-associated GM on hippocampal memory is, to the best of our knowledge, currently unknown. Here, we report that the transplantation of young (Y) mice with an age-associated GM, of both mouse and human origin, transfers some aspects of the deleterious impact of aging on the HPC, highlighted by a deficit in memory and an inability of the hippocampal network to respond to a novel environment. Furthermore, targeted pharmacogenetic manipulation of VN activity uncovers the role of VN tone on hippocampal function and its potential therapeutical impact on age-related hippocampal dysfunction.

## Results

### Age-associated GM, of both murine and human origin, impairs hippocampal functions.

**Figure 1:**
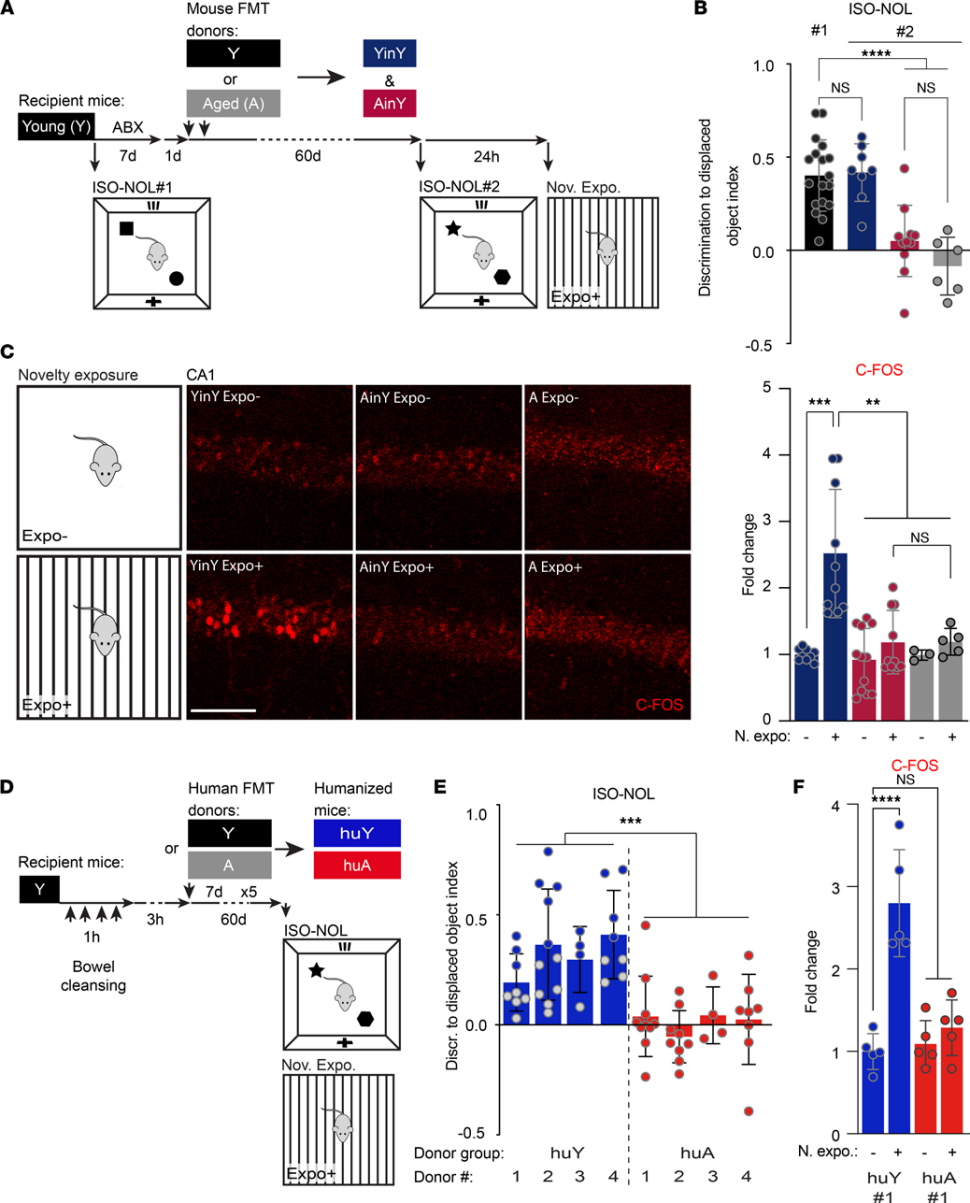
Age-associated gut microbiota (GM), of both murine and human origin, impairs hippocampal function and structure. (**A**) Timeline of the age-associated GM transplantation scheme in young (Y) adult mice (10- to 12-week-old). Y mice were ABX-treated for 7 days followed by fecal microbiota transplantation (FMT) from either young or aged (A) mice (18–20 months), to generate A mouse FMT in Y (AinY) mice and the corresponding Y mouse FMT in Y (YinY) control animals. The effect of the transplantation was assessed 60 days later by the measure of memory abilities in the isotropic version of the novel object location task (ISO-NOL), followed 24 hours later by the exposure to novelty assay. (**B**) Memory abilities in the ISO-NOL task of the Y mice prior to (ISOL-NOL #1) and after (ISOL #2) the age-related FMT in comparison to A mice (*n* = 18, 8, 10, and 6 mice per group). (**C**) Representative IHC images and quantitative analysis of the effect of A- versus Y-associated GM transplantation compared with aged mice on the number of C-FOS^+^ cells measured in CA1, after the exposure to novelty (*n* = 7, 8, 10, 7, 3, and 5 mice per group). (**D**) Timeline of the human GM-transplantation scheme, with the generation of the young human (huY) and aged (HuA) human donor FMT animals, and behavior in Y mice. (**E**) Memory abilities of the huA and huY mice in the ISO-NOL behavioral task (*n* = 4 donors per age human GM group, with 5–10 replicate mice per donor). (**F**) Quantification of the increase in the number of dorsal hippocampal CA1 C-FOS^+^ cells after the exposure to novelty in huA #1 *versus* huY #1 mice (*n* = 5 per group). Schematics (**A** and **D**) depict the experimental group color codes used for the associated quantifications. (**B**, **C**, and **F**) One-way ANOVA. (**E**) Two-way ANOVA with Fisher’s test. Data are shown as mean ± SD. **P ≤ 0.01; ***P < 0.001; ****P > 0.0001. Scale bars: 100 μm. FMT, fecal microbiota transplantation; n. expo., novelty exposure assay.

To investigate the potential contribution of the transplanted GM for the detrimental impact of aging on hippocampal functions, Y adult mice (10- to 12-week-old), hereafter referred to as Y mice, were treated with broad-spectrum antibiotics (ABX), followed by fecal microbiota transplantation (FMT). This method allowed for the transplantation of the recipient mice with the GM from Y or A — 18- to 20-month-old — donor mice to generate A mouse FMT in Y (AinY) mice and Y mouse FMT in Y (YinY) control animals (Figure 1A).

To verify the effectiveness of the ABX treatment to cleanse the mouse GM prior to FMT, we first analyzed its effect on GM bacterial content in a fecal microbiota (FM) plating experiment. Fecal samples were collected from the same mice before and after the ABX treatment. Results show this procedure to lead to an almost complete depletion in the fecal-related GM content in mice (Supplemental Figure 1; supplemental material available online with this article; https://doi.org/10.1172/jci.insight.147700DS1). Then, analysis of the microbial composition from the donors and FM transplanted mice was performed by 16S rRNA gene sequencing (Supplemental Figure 2). Changes in the relative abundance of bacteria in both donor and recipient mice were observed at the taxonomic level of family (Supplemental Figure 2A), with a trend toward an age-associated increase in the Firmicutes to Bacteroides ratio in donor samples that was no longer seen after FMT (Supplemental Figure 2B). The α diversity can be used to measure the richness and uniformity of species in community ecology. Using Shannon and Simpson indexes or Abundance-based Coverage Estimator (ACE), we did not observe significant changes in α diversity between Y, A, YinY, and AinY groups (Supplemental Figure 2, C–E). Determination of the beta diversity, the comparative analysis of the composition of the microbial community in different samples, showed differences in the GM from Y and A donors (clusters Y and A; Supplemental Figure 2F) and recipient Y mice receiving Y- or age-associated microbiota (clusters YinY and AinY; Supplemental Figure 2F). Although such changes were relatively subtle compared with previous studies using mice from different strains (2, 3, 8), comparative analysis of the Y versus A mice and transplanted animals showed a clustering due to the age of the donor that was transferrable to the colonized animals (Supplemental Figure 2F). This result is supported by the lower Jaccard distance between donor and recipient mice than between Y and A or YinY and AinY mice (Supplemental Figure 2G). Thus, we conclude that, even though the FMT approach used here led to a distinct microbial composition signature in each of the 4 different groups, some of the GM “aging” characteristics are transferrable to the recipient mice.

Next, the repercussion of GM colonization on hippocampal functions was investigated at the behavioral and cellular levels (Figure 1A). HPC-dependent memory ability was scored before and after GM colonization using the HPC-dependent isotropic version of the novel object location (ISO-NOL) task (20, 21), a task sensitive to aging (22). In this assay, Y mice showed normal discrimination memory performances in the test before FMT, and no changes were observed following GM transplantation from Y donors. Conversely, GM transplantation from A mice led to a deterioration of the recipient mice’s memory to levels similar to the scores seen in naive A mice (Figure 1B). The detrimental impact of the A mouse GM on Y recipient mice memory was not specific to this task, as similar deficits were also seen in other HPC-dependent behaviors — i.e., the novel object recognition (23) — in a version where the dorsal HPC implication was previously demonstrated in this task (24), and the contextual version of the fear conditioning (25), to a level of severity that matched the deficits seen in naive A animals (Supplemental Figure 3, A–C). Since the behavioral tasks used here rely on different HPC functions or connectivity networks — i.e., the HPC solely for the ISO-NOL task and HPC connection to the perirhinal and insular cortexes for the ISO NOR (23), and HPC to the amygdala and fear conditioning (25) — it seems that the age-associated GM negatively impacts the ensemble of HPC memory.

To examine the effect of the age-associated GM on the HPC neuronal network, the increases in the expression of the immediate early gene C-FOS following mouse exposition to a novelty exposure assay (26, 27) were quantified by automated counting in the dorsal hippocampal CA1 subregion (Figure 1C). This subregion was selected due to its known involvement in the memory task used here, an implication demonstrated through inactivation experiments or electrophysiological recordings (20, 28, 29). Novelty-induced upregulation of C-FOS in the dorsal CA1 was seen in animals colonized by a Y mouse GM, while mice receiving an age-associated microbiota showed reduced activation similar to that of A mice (Figure 1C). A comparable deficit in the density of C-FOS^+^ neurons in response to mouse exposition to novelty was also observed in the CA3 and dentate gyrus (DG) HPC subregions of age-associated GM transplanted mice (Supplemental Figure 3D).

To assess the potential impact of age-associated GM transplantation in more detail, we analyzed hippocampal astrogliosis and adult neurogenesis, 2 cellular processes highly sensitive to aging (30) and to GM composition (reviewed in ref. 31). As depicted in Supplemental Figure 3E, age-associated GM transplantation increases astrogliosis. It also significantly decreases adult hippocampal neurogenesis as reported by a reduction in the number of doublecortin-expressing (DCX-expressing) immature adult-born neurons in the DG (Supplemental Figure 3F). Thus, age-associated GM transplantation to Y animals impairs hippocampal memory, impairs the ability of the HPC network to respond to novelty exposure, promotes astrogliosis, and decreases the number of hippocampal newly generated neurons, mimicking important hallmarks of aging on hippocampal structure and function.

We then sought to investigate whether the transplantation of human GM could promote similar changes in the HPC of recipient mice. Fecal samples were collected from adult healthy human donors classified as Y (less than 35 years old) or A (more than 65 years old) (Supplemental Table 1) to generate A human donor FMT in A (huA) mice and Y human donor FMT in Y (huY) control animals (Figure 1D). Following this age-related human FMT into Y recipient mice, the potential effects on HPC-related memory were evaluated using the ISO-NOL task and the novelty exposure assay. Mice transplanted with a Y human GM performed normally in the task. In contrast, the microbiota transplantation from A human donors impaired HPC-dependent memory (Figure 1E) and prevented the novelty-induced CA1 activation (Figure 1F and Supplemental Figure 3G). Therefore, the microbiota transplantation from old, but not Y, human donors promoted alterations in hippocampal function in the recipient mice. Together, these data show that the age-associated GM, of both murine and human origin, plays an important role in the etiology of age-associated memory deficits.

### Age-associated GM negatively impacts ascending-VN signaling.

To gain some mechanistic insights on how the age-associated GM impacts HPC functions, we investigated the possible involvement of the VN in this process. Indeed, the VN is known to directly communicate with the brain through its NG neuron-related ascending signaling (reviewed in ref. 10), but the possible implication of the latter in the age-related deficits in HPC functions is poorly understood.

**Figure 2:**
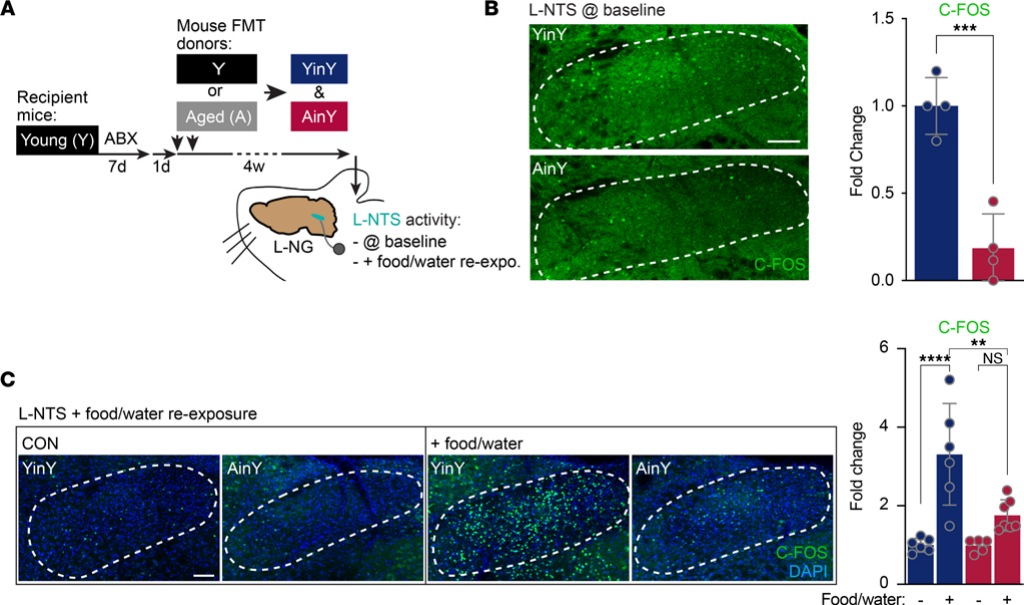
Impact of age-associated GM transplantation on ascending-VN signaling. (**A**) Timeline of the experiment with the experimental group color codes used for the associated quantifications. Four weeks following aged (A) or young (Y) mouse FMT in ABX-treated Y mice, C-FOS–based left-nucleus tractus solitarius (L-NTS) neural activity measurement was performed at either baseline or following food and water consumption using a food/water reexposition (food/water reexpo.) assay. (**B** and **C**) Effect of the age-associated GM transplantation on L-NTS neuronal activity level at baseline (mice in their home-cage) condition (*n* = 4 per group) (**B**) and following food and water consumption (*n* = 6, 6, 5, and 7 per group) (**C**). (**B**) One-tailed unpaired *t* test. (**C**) One-way ANOVA. Data are shown as mean ± SD. **P ≤ 0.01; ***P > 0.001. Scale bars: 100 μm.

First, the effect of the age-associated mouse GM transplantation in Y mice was investigated on VN ascending signaling into the brain (Figure 2A). The number of C-FOS^+^ neurons in the vmNTS was taken as a proxy of ascending-VN activity level, and this quantification was made in AinY and YinY mice at baseline (mice in their home cage) and following a food/water reexposure (food/water reexpo.) assay. The latter was used as a means to more directly engage VN ascending signaling. Automated quantification of NTS C-FOS^+^ cells revealed a reduction in neuronal activity in age-associated GM-transplanted mice compared with their Y mouse–associated GM-transplanted counterparts. This holds true under basal conditions (Figure 2B) and following VN activation through food and water consumption (Figure 2C). Such analysis reveals that age-associated GM transplantation leads to a deficit in VN ascending inputs to the brain, both at rest and following VN activation.

### Decrease in VN activity is both necessary and sufficient for the age-associated GM impact on the HPC.

**Figure 3:**
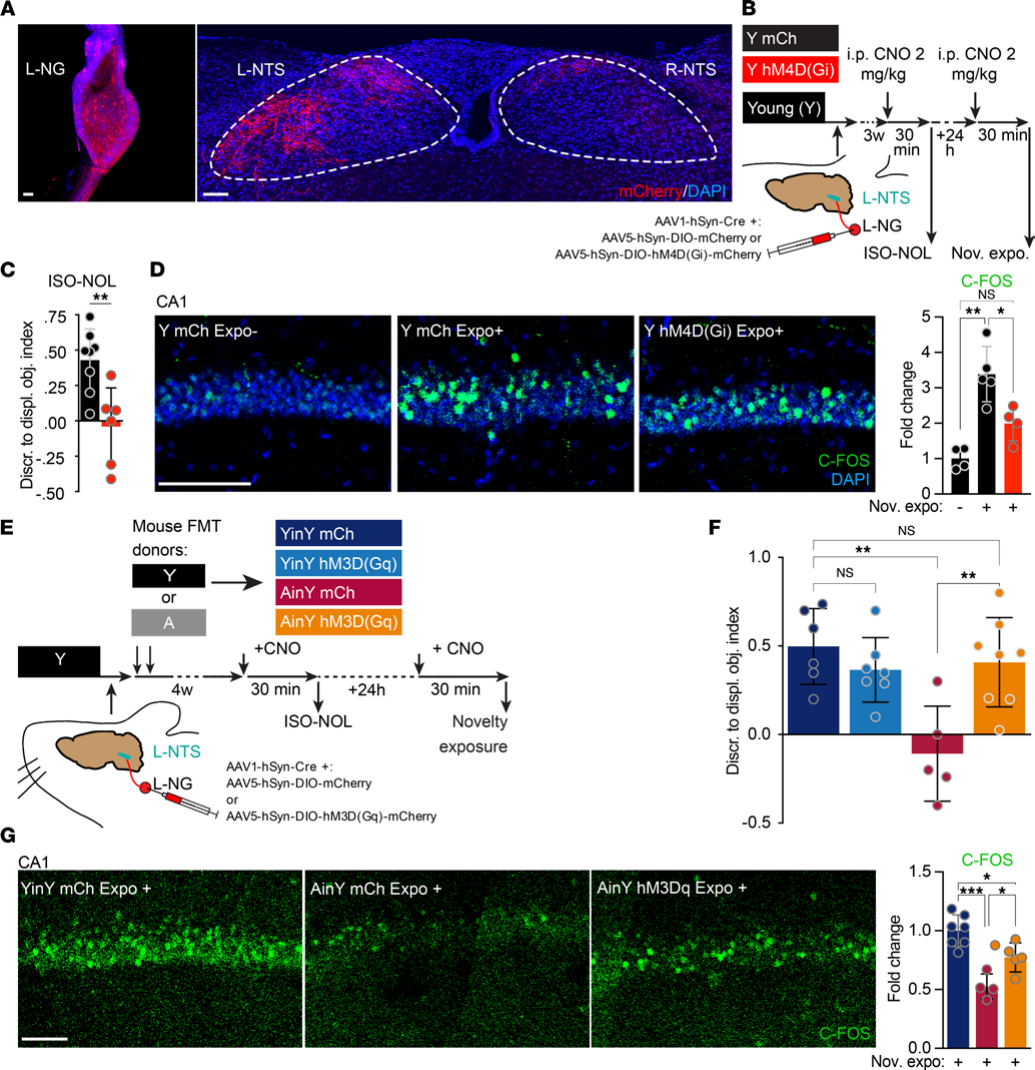
Decrease in VN signaling is necessary and sufficient for the age-associated GM’s negative impact on memory. (**A**) Representative image of an adeno-associated virus (AAV) mCherry transduced left nodose ganglia (L-NG) and of the corresponding ascending mCherry^+^ fibers at the level of the left and right nucleus tractus solitarius (L-NTS and R-NTS, respectively). (**B**) Timeline of the L-NG pharmacogenetic inhibition experiment. Young (Y) mice received a coinjection in the L-NG of a Cre-expressing virus with either a Cre-dependent inhibitory hM4D(Gi) DREADD (Y hM4D[Gi]) or a Cre-dependent mCherry (mCh) control virus (Y mCh). Three weeks after the L-NG injection of viral vectors, animals were i.p. injected with clozapine N-oxide (CNO) 30 minutes prior to the isotropic-novel object location (ISO-NOL) task and novelty exposure (nov. expo.) assay. The 2 tests were performed 24 hours apart. (**C** and **D**) Effect of a L-NG DREADD inhibition on the memory ability in the ISO-NOL task (*n* = 8 and 6) (**C**) and the increase in the number of dorsal hippocampal CA1 C-FOS^+^ cells (**D**) after the exposure to novelty (*n* = 4, 5 and 4 per group), in Y hM4D(Gi) versus Y mCh control mice. (**E**) The timeline and procedure of the L-NG pharmacogenetic activation in AinY mice experiment and assessment of its effect in the ISO-NOL and novelty exposure assay was identical to the previous inhibitory DREADD experiment but with the usage of the activatory hM3D(Gq) DREADD in place of the inhibitory version. L-NG injection of viral vectors were directly followed by an age-associated FMT to generate L-NG transduced/FMT animals (AinY hM3D[Gq], YinY hM3D[Gq], AinY mCh, and YinY mCh). (**F** and **G**) Effect of the pharmacogenetic vagal activation in AinY mice on the memory abilities in the ISO-NOL task (*n* = 6, 7, 5 and 8) (**F**) and the increase in the number of dorsal hippocampal CA1 C-FOS^+^ cells (**G**) after the exposure to novelty in YinY and AinY mCh– versus AinY hM3D(Gq)–expressing mice (*n* = 7, 4, and 5 per group). Schematics (**B** and **E**) depict the experimental group color codes used for the associated quantifications. (**C**) One-tailed unpaired *t* test. (**D**, **F**, and **G**) One-way ANOVA. Data are shown as mean ± SD. *P < 0.05; **P > 0.01; ***P > 0.001. Scale bars: 100 μm. DIO, double-inverted opsin.

Next, to directly test the possible implication of VN activity for hippocampal memory function, we manipulated VN ascending activity by viral transduction of the designer receptor exclusively activated by a designer drug (DREADD) in the left nodose ganglia (L-NG). This side was chosen to be consistent with the clinical setup used for electrical VN stimulation as a treatment for resistant major depression (32). A Cre-expressing virus and a conditional Cre-dependent virus expressing the mCherry-coupled (mCh-coupled) excitatory hM3D(Gq) DREADD were coinjected in the L-NG of Y mice (Supplemental Figure 4A, group 2). Three weeks after viral delivery, efficiently labeled L-NG neuron soma and their axonal terminals could be detected in the ventromedial areas of the ipsilateral NTS (Figure 3A). At that same time, the DREADD activator clozapine N-oxide (CNO) was i.p. administered to drive the DREADD activation (Supplemental Figure 4A). Three hours later, this treatment significantly increased the number of C-FOS^+^ neurons in the vmNTS of hM3D(Gq) transduced animals (group 2), compared with control mCh-only (group 1) transduced animals. Furthermore, the ability of the DREADD approach to inhibit ascending-VN signaling was also tested. To do so, animals were L-NG virally cotransduced with an hM3D(Gq) virus combined with an excess of hM4D(Gi) inhibitory form of DREADD (Supplemental Figure 4A, group 3). CNO treatment of the cotransduced animals showed a drastic reduction in the ipsilateral vmNTS neuronal activation, compared with the same CNO treatment in hM3D(Gq) transduced animals (group 2), to levels virtually indistinguishable from control CNO-injected mCh-only (group 1) transduced animals (Supplemental Figure 4, B and C). This result demonstrated that the experimental procedure used here enables both activatory and inhibitory pharmacogenetic control of VN ascending signaling.

We next evaluated the hippocampal memory strength following a transient inhibition of the vagal ascending activity, thus mimicking the age-associated GM effect on ascending vagal signaling. For this purpose, 3 weeks after the viral delivery, L-NG hM4D(Gi) transduced Y mice were generated (Y hM4D[Gi] mice and Y mCh control animals) and were given a CNO i.p. treatment 30 minutes prior to the ISO-NOL task and novelty exposure assay in order to induce a transient inhibition of the targeted ascending-VN circuit during both experimental procedures (Figure 3B). L-NG inhibition during the ISO-NOL test completely impaired mice’s discrimination ability in the task (Figure 3C), and this loss was associated with an absence of novelty-induced increase in C-FOS^+^ cell number in the dorsal CA1 region (Figure 3D). We next investigated whether a stimulation of ascending-VN activity could counteract the age-associated GM deleterious effect on HPC functions. To test this possibility, mice received a viral injection in the L-NG to express the excitatory hM3D(Gq) DREADD before being submitted to an age-associated FMT. First, the effectiveness of the pharmacogenetic VN activation in AinY mice was verified (Supplemental Figure 4D). CNO injection in L-NG hM3D(Gq) transduced AinY mice resulted in an effective L-NTS neuronal activation in comparison with mCh AinY and YinY control animals (Supplemental Figure 4E), demonstrating the robustness of the protocol used to activate the ascending-VN pathway, irrespectively of the transplanted GM donor’s age. A similar procedure of CNO-based activation of L-NG ascending signaling in hM3D(Gq) transduced AinY and YinY mice prior to the ISO-NOL memory task (Figure 3E) showed that hM3D(Gq) transduced YinY (YinY hM3D[Gq]) mice were not affected by this transient ascending-VN signaling activation, while AinY hM3D(Gq) mice depicted a complete rescue of their memory abilities, when compared with AinY mCh and YinY mCh control animals (Figure 3F). Thus, pharmacogenetic VN stimulation can reinstate normal cognitive status in AinY mice. At a cellular level, a CNO-mediated DREADD ascending-VN stimulation 24 hours later (Figure 3E) increases hippocampal C-FOS induction following the animal exposure to novelty in AinY hM3D(Gq) mice when compared with AinY and YinY mCh control animals (Figure 3G). The latter result shows that the increase in ascending-VN signaling rescues the HPC ability to respond to novelty in AinY mice. Collectively, these data reveal that inhibiting the activity of ascending-VN activity is necessary and sufficient for the detrimental impact of age-associated GM transplantation on HPC memory.

### Activation of ascending-VN signaling is beneficial to A mouse memory.

**Figure 4:**
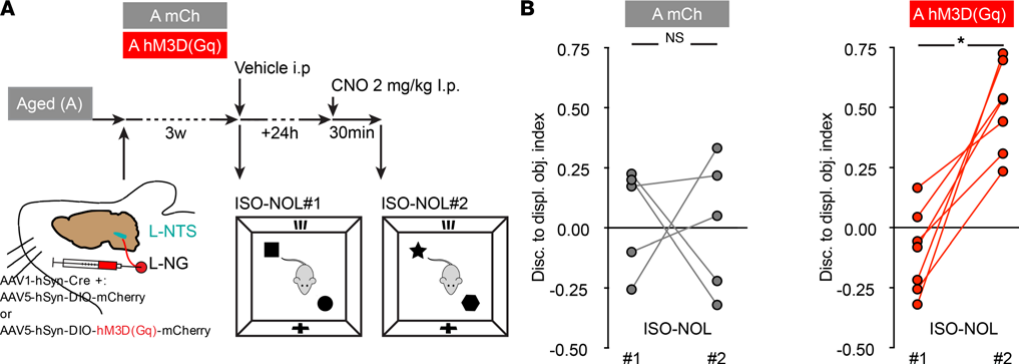
Activation of VN ascending signaling increases the memory abilities of aged mice. (**A**) Timeline of the experiment and schematic of the experimental group color codes used for the associated quantifications. Aged (A) mice received a coinjection in the left-nodose ganglia (L-NG) of a Cre-expressing virus with either a Cre-dependent activatory hM3D(Gq) DREADD (A hM3D[Gq]) or a Cre-dependent mCherry (mCh) control virus (A mCh). Three weeks following the viral delivery, memory was assessed 30 minutes after an i.p. CNO-vehicle injection (ISO-NOL #1), followed 24 hours later by a clozapine-N oxidse (CNO) i.p. injection (ISO-NOL #2). (**B**) Measure of memory abilities in ISO-NOL #2 versus ISO-NOL #1 of A hM3D(GQ) mice (*n* = 7) compared with A mCh (*n* = 5) animals. Two-tailed Student’s *t* test with Wilcoxon matched-pairs signed rank test. Data are shown as mean ± SD. *P < 0.05. AAV, adeno-associated virus; DIO, double inverted opsin.

Finally, we reasoned that since age-associated GM inhibition of VN ascending signaling was causal to the detrimental impact of aging on memory, restoration of its activity should also restore memory of A mice. This hypothesis was tested by performing the transient ascending-pharmacogenetic VN activation procedure in A mice (Figure 4A). This manipulation indeed increased the memory ability of CNO-activated hM3D(Gq) transduced A mice (A hM3D[Gq]) in the ISO-NOL test, in comparison with levels of the same animal at baseline (prior day with no CNO injection), whereas no CNO-dependent memory enhancement was seen in control mCh-only VN transduced A (A mCh) animals (Figure 4B). These findings open potentially new therapeutic approaches aimed at acting either directly on the GM composition or on VN activity to alleviate the detrimental impact of aging on memory.

## Discussion

Our study unravels a contribution of age-associated GM — from both murine and human origins — in mouse hippocampal alterations similar to those observed with aging. It also highlights the participation of the VN in mediating this age-related phenomenon through the gut-brain axis (Supplemental Figure 5). To translate the effect of an acute pharmacogenetic modulation of NG ascending activity to the HPC, a neural transduction pathway is likely to take place. Indeed, given the temporal dynamic, the neural route seems more probable, as opposed to the humoral and/or immunity-based routes of communication. It is particularly interesting to note that this acute pharmacogenetic ascending-VN activation can reinstate normal memory in age-associated GM transplanted and A mice, despite the presence of glial fibrillary acidic protein–based (GFAP-based) signs of HPC neuroinflammation in these mice. We postulate that an increase in VN ascending signaling might counteract the neuroinflammation to reinstate a normal hippocampal function. This neural pathway hypothesis is consistent with the fact that the VN connects both the locus coeruleus and dorsal raphe, which release NA and 5-HT, respectively (16, 17). The VN also exerts a modulation in activity of the basal forebrain area (33), which releases acetylcholine (Ach) to the entire forebrain, including the HPC. Therefore, a deficit in ascending NG could lead to a reduction of those neurotransmitters’ release in the HPC. This would lead to a deficit in the general activity level in the HPC that would translate into a deficit in memory abilities. This hypothesis supports the decrease in neuronal activity level seen in the HPC of age-associated GM transplanted animals in the novelty exposure assay used here. This NTS/basal forebrain modulation of activity was recently illustrated with the description of the NTS-septum-HPC circuit regulation of hippocampal-dependent memory (18). The fact that a stimulation of VN ascending signaling is effective in counteracting the detrimental impact of both (a) the age-associated GM transplantation and (b) aging raises the question of the specificity of this procedure in regard to aging. This matter, despite being beyond the scope of our study, could be addressed in future research notably through testing the effect of the pharmacogenetic-mediated increase in VN ascending signaling in some microbiota-related contexts distinct from aging, like in a model of ABX-driven hippocampal memory dysfunction (4) or microbiota-independent paradigms such as acute pharmacologic retrograde amnesia (34). As for the potential neuronal circuit involved in translating the age-associated GM effect through the vagal/NTS system to the HPC described herein, this communication route must be multisynaptic. Indeed, the vmNTS, where the VN projects its terminals, connects to many brainstem and forebrain regions, but not to the HPC directly (35, 36). Besides the possible involvement of the NTS-septum-HPC circuit mentioned above (18), another possibility is the recently described connection of the locus coeruleus with the HPC through dopaminergic connections (37). This brain structure is known to be one of the main relays of vagal activity, and its dopaminergic projections to the HPC were recently described to be prominent in the neural encoding of novelty by the HPC (37). This function and circuit are most likely important in the detection of novelty in the hippocampal memory tasks used here, such as the NOL memory test, and the possible involvement of a decrease in locus coeruleus–dopaminergic (LC-dopaminergic) inputs to the HPC through the age-associated GM downregulation of VN ascending signaling could be investigated in the future. Our findings open therapeutic avenues aimed at acting either directly on the GM composition or on VN activity to alleviate the detrimental impact of aging on memory.

## Methods

Supplemental Methods are available online with this article.

### Statistics

All experiments and data analyses were achieved in a blinded fashion. Each experiment was replicated at least twice. Statistical analyses were performed with GraphPad Prism 8.0. No statistical methods were used to pre-determine sample size, or to randomize samples. Prior to further analysis, sample normality was tested using the D’Agostino and Pearson omnibus normality test. For statistical analysis, between 2 groups, 1-tailed unpaired *t* test, with an assumption of equal variance, was used and between 3 or more independent groups; 1-way ANOVA tests coupled to post-hoc Sidak’s multiple comparison tests was used. In Figure 1E statistical analysis, 2-way ANOVA with Fisher’s test was used. In behavioral experiments, outliers were identified using Grubbs’ method (α = 0.05) and then removed. Figure legends indicate the number of subjects used in each experimental condition and the methods of statistical analysis. Data are expressed as mean ± SD. Statistical significance was set at *P < 0.05, **P < 0.01, ***P < 0.001, ****P < 0.0001.

### Study approval

#### Humans.

FM donors that matched the Y and A category were selected from healthy volunteers through the ICAReB platform from the center for translational science, Institut Pasteur (38). All participants received oral and written information about the research and gave written informed consent in the frame of the healthy volunteers Diagmicoll cohort (clinicaltrials.gov, NCT 03912246) after approval of the CPP Ile-de-France I Ethics Committee (April 30, 2009) and CoSImmGEn cohort (clinicaltrials.gov, NCT 03925272), after approval of the CPP Ile-de-France I Ethics Committee (January 18, 2011).

#### Animals.

experiments were performed using adult (10- to 12-week-old) male RjOrl: SWISS mice purchased from Janvier labs (St. Berthevin, France). Animals were housed under a 12-hour/12-hour light/dark cycle, with dry food pellets and water accessible ad libitum, at the Pasteur Institute animal care facility, officially registered for experimental studies on rodents. All animal experiments were designed according to the European community council directive of November 24, 1986 (86/609/EEC) and the European Union guidelines, to the 3R’s rules and were supervised by the French Ministry of Research, as well as reviewed and approved by the Animal Welfare Committee of the IP (project no. 2016-0023) and the “Service prevention des risques” (experimental protocol 17.029).

## Author contributions

DR designed, developed, and managed the project; performed all experiments; and wrote the manuscript, under the supervision of PML. HS performed 16S rRNA gene sequencing analysis, with the help of SS. MH and AHR were involved in behavioral and histological analysis. The gnotobiology center, under the supervision of MB, was involved in the setup and realization of mice FMT experiments. The ICAReB platform, and particularly BLP, handled the donor sample regulatory and logistic aspects of the project, under MNU’s supervision.

## Supplementary Material

Supplemental data

## Version 1 06/23/2022

In-Press Preview

## Version 2 08/08/2022

Electronic publication
