# Supplementary material for: Age-associated gut microbiota impair hippocampus-dependent memory in a vagus-dependent manner
Source: JCI Insight. 2022 Aug 8;7(15):e147700. doi: 10.1172/jci.insight.147700 (PMC9462480; doi:10.1172/jci.insight.147700)
Supplement: Supplemental data [file jciinsight-7-147700-s174.pdf]

Supplementary figures

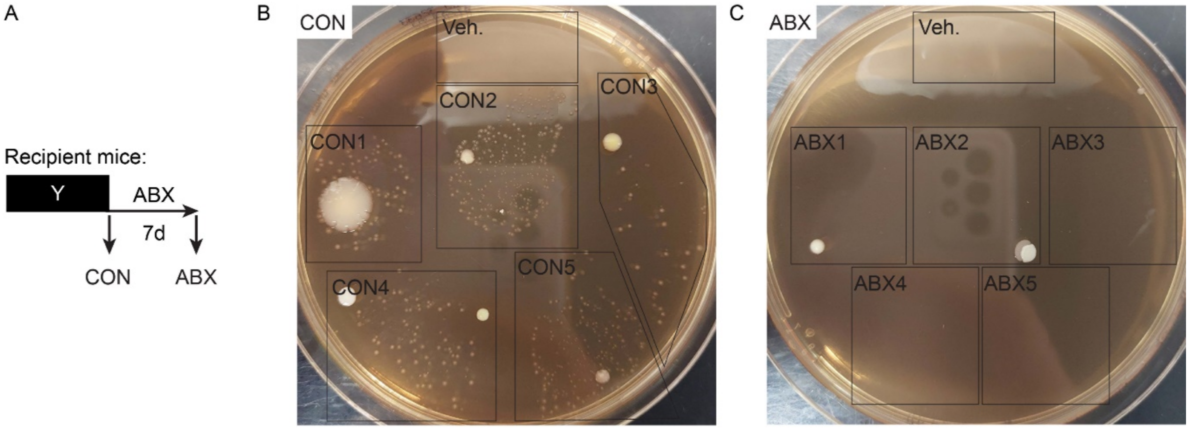

Fig. S1-1. Efficiency of the antibiotic (ABX)-based GM cleansing prior to FMT. (**A**) Timeline of the experiment to assess the efficiency of the pre-FMT large spectrum ABX-based GM cleansing procedure. Bacterial growth from FM resuspension from the same mice before (**B**), and after (**C**), receiving the ABX treatment.

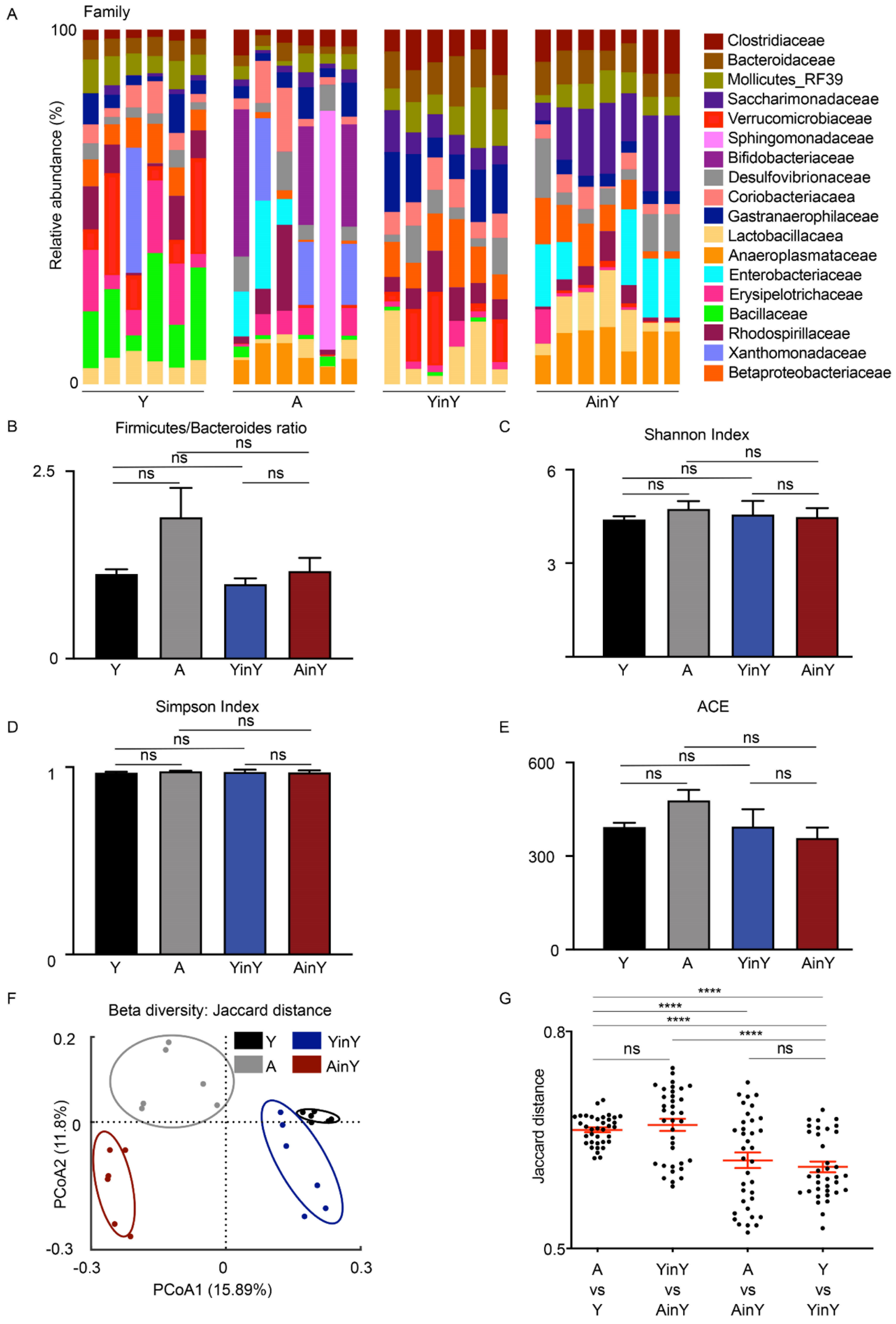

Fig. S1-2. 16S-based characterization of the age-associated mouse GM-transplantation to young mice model.

(**A**) Bacterial microbiota composition at the family level in experimental conditions of young (Y) and aged (A) FM-donors and FMT mice (YinY and AinY). (**B**) The Firmicutes-Bacteroides ratio between the experimental groups show no significant changes. Shannon (**C**) and Simpson (**D**) index of species diversity, and abundance-based Coverage Estimator (ACE) (**E**), showed non-significant differences between the four experimental groups. (**F**) Principal coordinate analysis (PCoA) of Jaccard distance among samples from the four experimental groups, where PCoA1 and PCoA2 represented the top two principal coordinates that captured most of the diversity. The fraction of diversity captured by each coordinate is given as a percentage. (**G**) Jaccard distance between samples of indicated experimental groups. (B-E, G) One-way ANOVA. Throughout the figure, bars represent the mean ± SD. N.s., non-significant and ****$p < 0.0001$.

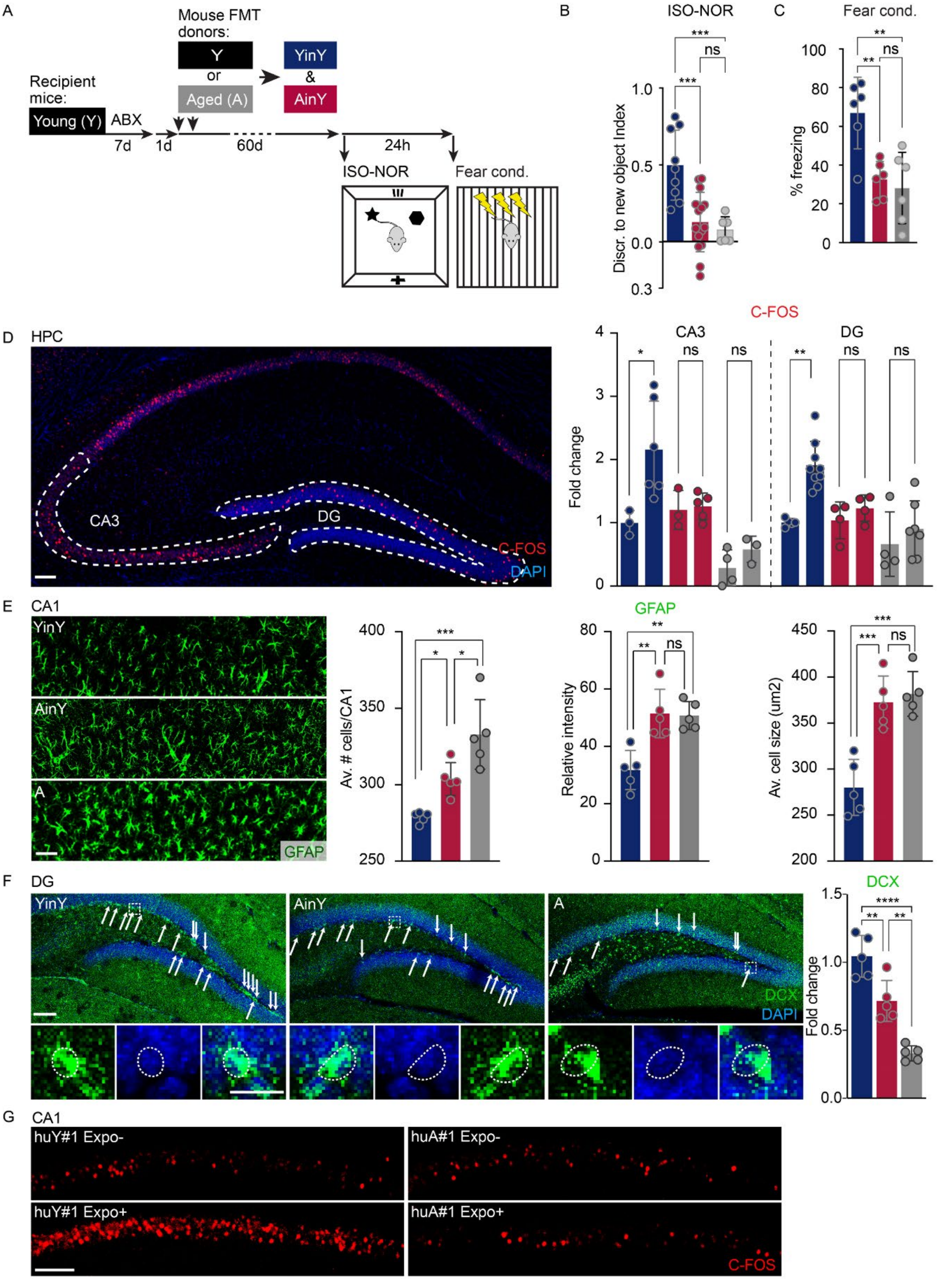

Fig. S1-3. Age-associated GM impairs hippocampal (HPC) function in the contextual fear conditioning and isotropic version of the novel object-recognition (ISO-NOR) tasks. (**A**) Timeline of the age-associated fecal microbiota transplantation (FMT) scheme in young (Y) mice, with the generation of the Y mice FMT in Y (**YinY**) mice or aged (A) mice FMT in Y (**AinY)** mice, and of its effect on memory abilities of the transplanted mice in, (**B**) the ISO-NOR task (n = 9 and 15 mice per group), and (**C**) a contextual fear conditioning (fear cond.) task (n = 6 per group). (**D**) Representative immunohistochemical images of a dorsal HPC from a YinY novelty exposed animal and quantitative analysis of the effect of age, versus young, -associated GM transplantation compared to

A mice on CA3 and dentate gyrus' (DG) increase in the number of C-FOS-positive cells after the exposure to novelty assay (n = 3, 6, 3, 5, 4, 3 for CA3 and 4, 9, 4, 4, 4 and 7 for DG mice per group). Effect of the age-associated GM on, (**E**) CA1 glial fibrillary acidic protein (GFAP)-astrogliosis related measures (n = 5 per group), and (**F**) the number of DG doublecortin (DCX) positives cells (n = 5 per group; arrows point to positive cells and the insets a representative image of a DCX positive cell in each group). (**G**) Representative images of the effect of human age-associated GM transplantation on increase number of C-FOS-positive neurons in CA1 after the exposure to novelty assay. Schematic (A) depicts the experimental group color codes used for the associated quantifications. (B-E) One-way ANOVA. Throughout the figure, bars represent the mean ± SD. N.s., non-significant; $p > 0.05$; *$p < 0.05$; **$p < 0.01$; and ***$p < 0.001$. Scale bars, 100 μm throughout the figure except insets in (F), 20 μm.

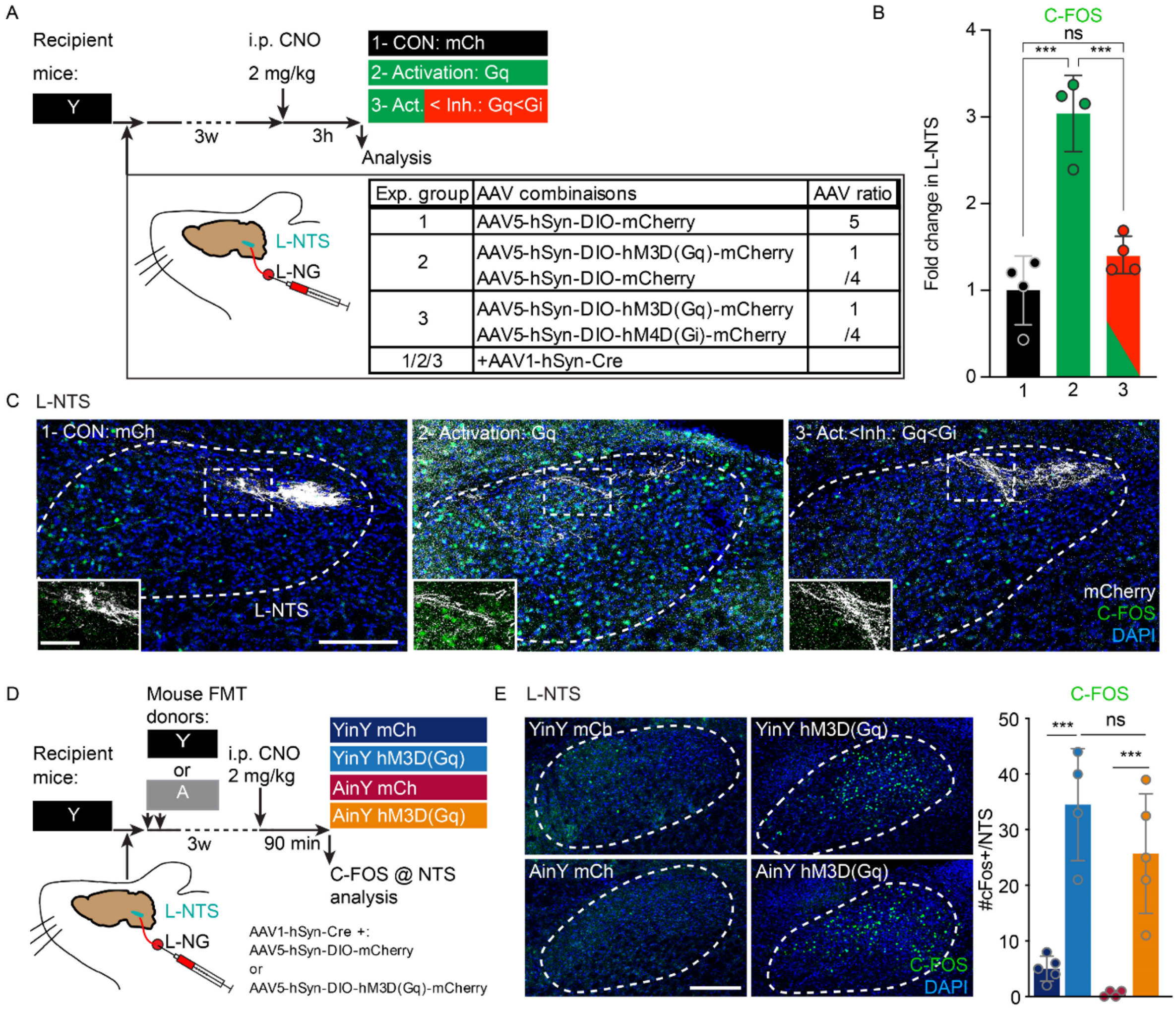

Fig. S2. Ascending left-nodose ganglia (L-NG) DREADD-based viral approaches efficiently promote both inhibition and activation in the ipsi-lateral nucleus tractus solitarius (NTS), and, for the latter, irrespectively of the GM-transplantation donor mice age group. (**A**) Timeline of the strategy to assess the potential of the viral and pharmacogenetic-based strategy to modulate ascending VN-signaling. Young (Y) mice received an injection in the L-NG of a Cre-expressing virus combined with either a Cre-dependent mCherry (mCh) virus (Exp. Group: 1 = **CON: mCh**), or two Cre-dependent activatory hM3D(Gq) DREADD and mCh viruses (Exp. Group: 2 = **Activation: Gq**), or two Cre-dependent hM3D(Gq) and hM4D(Gi) viruses (Exp. Group: 3 = **Act.<Inh.: Gq<Gi**). For the latter condition, hM4D(Gi) was in a 4 to 1 excess compared to hM3D(Gq). Three weeks after L-NG injection of viral vectors, animals were intraperitoneally (i.p.) injected with clozapine N-oxide (CNO) three hours prior to analysis. (**B**) quantification of the effect of L-VN hM3D(Gq) activation and of the potential of hM4D(Gi) to counteract a VN ascending activatory effect, through quantification of the number of C-FOS positive cells in the ipsi-lateral NTS of CNO treated Gq and Gq<Gi mice compared to mCh animals (n = 4 per group) and (**C**) representative images of L-NG mCherry-positive ascending fibers and C-FOS positive cells in the L-NTS of the corresponding animals. (**D**) Timeline of the viral and pharmacogenetic-based strategy to verify the effectiveness of L-NG activation in age-associated GM transplanted mice. L-NG injection of viral vectors, were directly followed by an A or Y mice fecal microbiota transplantation (FMT) to generate L-NG transduced AinY (**AinY hM3D(Gq)**) mice and the corresponding **AinY mCh**, **YinY hM3D(Gq)** and **YinY mCh** control animals. Representative images and quantification (**E**) of the effect of the pharmacogenetic vagal activation in AinY mice on the number of C-FOS positive cells in the ipsi-lateral NTS (n = 4, 4, 4 and 5 per group). Schematic (A) and (D) depict the experimental group color codes used for the associated quantifications. (B, E) one-way ANOVA. Throughout the figure, bars represent the mean ± SD. N.s., non-significant; ***$p < 0.001$. Scale bars, 100 µm, except in inset of C = 50 µm. Abbreviations: CON, control; Act., activation; Inh., Inhibition; AAV, adeno-associated virus; DIO, double inverted opsin.

Fig. S3. Graphical conclusion

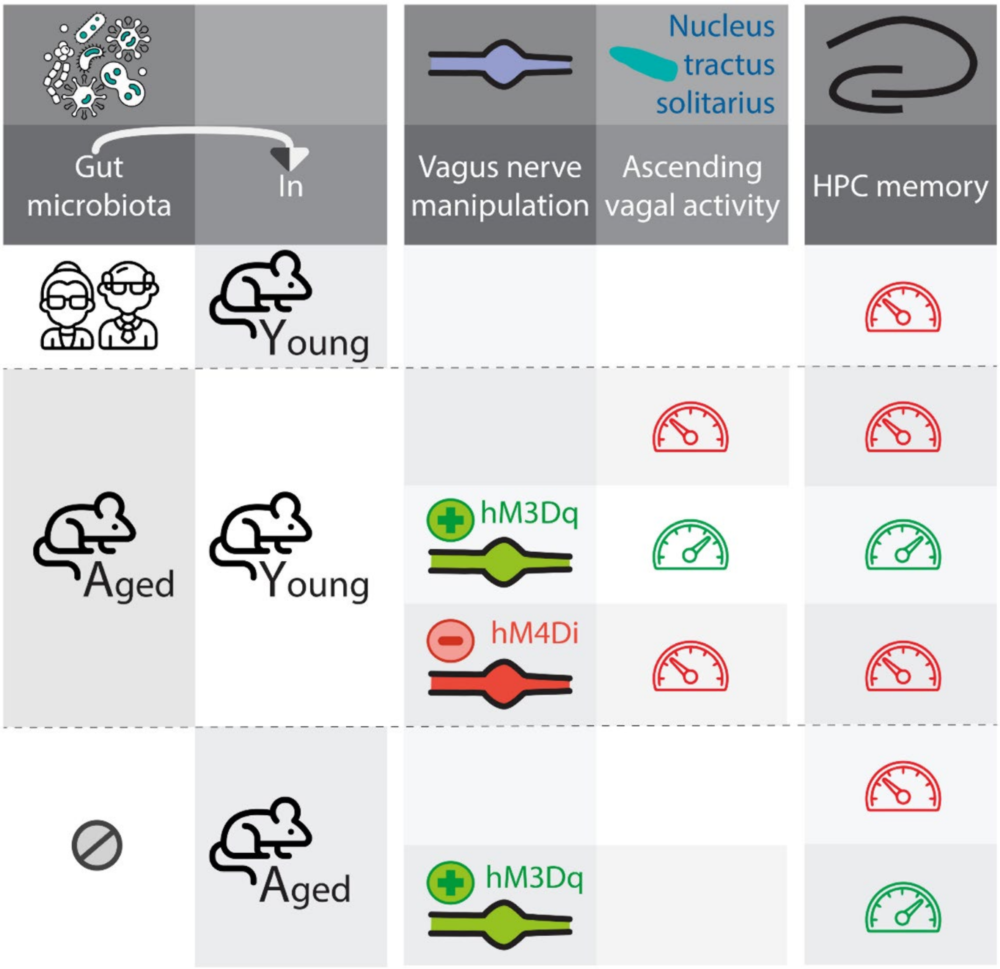

Supplementary methods

Fecal microbiota transplantation. Prior to transplantation, microbiota-recipient mice received a broad-spectrum antibiotic mixture treatment of ampicillin (1 mg/ml), streptomycin (5 mg/ml) and colistin (1 mg/ml) in drinking water for 7 consecutive days combined with oral gavage with a solution of vancomycin (0,1 mg/ml) and metronidazole (0,5 mg/ml) on day 3 and 5 of the antibiotic treatment. The latter was ceased 24h before the microbiota transplantation by returning mice to regular drinking water. Fresh fecal samples (1 g in 5 mL sterile PBS) were used for the transplantation protocol, harvested from either young (Y) or aged mice (A). Recipient mice received the fecal suspension (300 mL per mouse) by oral gavage at 1- and 3-days following antibiotics discontinuation, and were maintained in isolators from one day prior to it and up to the onset of fecal matter harvest and of behavioral testing 6 and 7 weeks later, respectively.

Effectiveness assessment of the pre-FMT antibiotic-based GM cleansing procedure. Two fecal pellets were harvested from the 5 same mice, before and after the 7 days broad spectrum antibiotic treatment, directly in sterile 16% glycerol PBS. Samples were homogenized using 1ml plastic syringe and centrifugated for 3 min at 13.000g. Supernatant were transferred to a new tube and stored at -80°C. Samples were thawed and 5 ul of this GM resuspension were mix in 100ul of sterile PBS, before being plated on a TGV-agar medium plate and incubated at 37°C for 3 days. Vehicle condition consisted in using the 16% glycerol PBS solution in place of the GM resuspension and was plated alongside the mice samples, to verify for the sterility of the solutions and procedure.

Mice fecal harvest, DNA extraction, amplicon sequencing and 16S rRNA gene sequence data analyses. 6 weeks following FMT, fecal material was harvested at 9 am and stored at -80C until processing. Human donors and 6 weeks-post FMT mice fecal material was used for microbiota profiling. DNA was extracted from fecal samples using 3 mice feces and 150 mg of human material, using the RNEasy PowerMicrobiome (Qiagen) according to the manufacturer's instructions for nucleic acid extraction with the only adaptation being a 15 min vortexing for the sample lysis step. DNA concentration was normalized to 5 ng/µL by dilution with DNA elution solution (Qiagen) to produce a final volume of 20 µL. Normalized DNA samples were sent to the institute Pasteur BIOMICS center for PCR amplification of 16S RNA genes and paired-end Illumina sequencing (2 × 250 bp) on the MiSeq platform. The V4 hypervariable region of the 16S rRNA genes was amplified using the S-D-Bact-0341-b-S-17, and S-D-Bact-0785-a-A-21 primers from (39). Following DNA extraction and sequencing, raw paired-end reads were processed in a data curation pipeline, that includes a step of removal of low-quality reads (Qiime2 2019.10) (40). Remaining sequences were assigned to samples based on barcode matches, and barcode and primer sequences were then trimmed. Sequences were denoised using the DADA2 method, and reads were classified using Silva reference database (version 132) (41). Alpha and beta diversity were computed using the phyloseq package (v1.24.2) (42) in R version 3.6.2. Principal Coordinate analyses of the Jaccard index were performed to assess beta diversity. The number of observed species, Chao1, Shannon and Simpson indexes were calculated using rarefied data (depth = 23,000 sequences/sample) and used to characterize alpha diversity. Raw sequence data are accessible in the European Nucleotide Archive (accession number: 2021-0023). Differential analysis was performed using the linear discriminant analysis effect size (LEfSe) pipeline (43).

Isotropic novel-object location/recognition and fear conditioning task procedure. Procedure was run as described with minor modifications. Briefly, 7 weeks after initiation of the FMT procedure, behavior was run in the morning, mice were transferred to the testing room at least 1 h before testing. Behavioral arenas were 44 cm sides squares in which animals were habituated for 10 min per day for two consecutive days. The next day a same duration object training phase was initiated for each animal with the following objects: for ISO-NOL#1, a 31,8/15,8/9,6 mm yellow duplo brick (Lego) and a 50 ml falcon blue lid (Falcon), for ISO-NOL#2, a 35 mm diameter/40 mm high black plastic cylinder and a 40/30/20 mm aluminum rectangular cube. In both cases, the two objects were placed in the two same side of the arena-corners, 6cm away from both vertical arena walls. It was followed by a 6 min testing phase were one of the objects (counterbalanced between the different animals) was moved to the opposite, same side corner of the arena (see schematic Fig. 1A). Four arenas were used with the same number of animals ran at once. For all stages where animals were in contact with the objects, the exploratory behavior was recorded and intertrial interval was 1 hour. ISO-NOL#1 and #2 tests were run prior and 60 days following the start of the antibiotic treatment, respectively. Videos were analyzed by an experimenter blinded to the experimental treatment. Mice were scored as exploring an object if they showed obvious signs of directed attention (i.e., sniffing or contact). Time spent on top of the objects was not counted, unless the mice were simultaneously directing attention to the object. The discrimination index was calculated from by dividing the difference in the object exploration time between the familiar and new location objects with the sum of the time exploring both objects. ISO-NO and fear conditioning task was run as previously described (44).

Novelty-exposure assay. Animals were exposed to a novel context (an arena with a textured plastic floor) for 3 minutes, before being returned to their home cage. 90 minutes after this novelty exposure, a time corresponding to the pick of C-FOS expression following a behavioral stimulus, mice were intracardially perfused with paraformaldehyde, their brains harvested and the number of C-FOS positive cells in the hippocampal CA1 subregion was quantified. For the latter, automated counting was done with the ICY software (Institut Pasteur, France Bioimaging) and its 'spot detection' function in brain areas (CA1, CA3 or DG) previously drawn on the slides' images via the region of interest function. Parameters of the spot detector were set on control condition images to match the detection made manually in parallel. The same parameters were then run on all experimental conditions images and allowed an unbiased automated detection.

Subject recruitment and human GM sample collection. Recruitment of young and aged healthy subjects were recruited on the basis of being, penally responsible (>18 years old) at the time of inclusion, drug naïve, and either less than 35 (young adult subject group) or more than 65 (aged subject group) years old, having an absence of systemic or familial history of medical illness or mental disorders and of antibiotics treatment less than a month before the sample collection. Table 1 depicts the age and sex information of the human donors involved in the study.

Human donor ID	Sex	Age	Group
816-199	M	22	Young
816-099	M	28	
1006-B-1-111	M	31	

1006-B-1-138	F	34	
816-138	M	67	Aged
1006-B-1-009	M	73	
816-161	M	63	
1006-B-1-008	F	71	

Table 1: FM human donor information.

Sample collections were made following the standard operating procedure IHMS_SOP 003 V2 for fecal samples self-collection and laboratory analysis handled within 4 hours from the International human microbiome initiative with minor modification (45) http://www.microbiome-standards.org/index.php?id=Sop&num=003). Briefly, samples were self-collected by donors in the morning in 120 ml urinary samples collection container (Dutscher, 861086) modified to have the lid pierced to allow for gas exchange, immediately moved to an oxygen chelating plastic pouch system (Biomerieux, Genbag Anaer 45534) with the oxygen chelating pad added and sealed according to instruction of the manufacturer. The ensemble was moved to a secondary container previously stored at 4°C, containing a calorimetric cold pack to ensure a temperature control of the samples and was brought for processing less than 3 hours post-collect. Samples were aliquoted for 16S rRNA gene sequencing and for human FMT to mice, following a previously designed protocol (46), and stored at 80°C until use.

Immunohistochemistry. Mice were euthanized by an intra-cardiac infusion of 0.9% NaCl followed by 4% Formol under deep anesthesia (xylazine 20 mg/kg, ketamine 100 mg/kg). Brains were extracted and post-fixed overnight with Formol 4% at 4°C, received three 10-minutes PBS washes before being coronally cut into 50 µm thick sections using a vibratome (Leica), and then stored at -20°C in cryoprotective solution (27,5% sucrose, 27,5% ethylene glycol, 45% PBS). Prior to immunostaining, sections went through three 10-minutes PBS washes. The blocking buffer used to permeabilize/block and incubate the sections with the primary and secondary antibodies was a mixture of PBS with 0.2% Triton X-100 and 10% Normal Donkey Serum (Abcam, Ab7475) and Na-azide 0.01%. Immuno-labelling consisted in a blocking/permeabilization step of 1h30 under constant agitation, then primary antibodies were incubated overnight (48h for C-FOS) at 4°C under constant agitation. Primary antibodies used were: C-FOS (1:4000, rabbit, Sysy, 226 003; 1:2000; guinea-pig, Sysy, 226 005), GFAP (1:2000, chicken, Abcam, ab13970), DCX (1:1000, rabbit, Abcam, Ab62341), mCherry (anti-RFP, 1:4000, rabbit, Rockland, 600-401-379). After three 5-minutes washes with PBS, sections were incubated for 1h30 under agitation with either Alexa Fluor 488 or 568–conjugated secondary antibodies followed by a 5-minutes DAPI (0.001% in PBS) counterstaining wash followed by two 5-minutes PBS washes, at room temperature. Sections were then mounted on slides with a mounting medium (FluoroMount-G, Interchim). Image acquisition was carried out using a confocal microscope (LSM 700, ZEISS).

Food/water re-exposure assay. In the morning food/water were given to mice previously food/water fasted for 15h. Two hours later, mice were intracardially PFA perfused and the effect of the treatment on the L-NTS-C-FOS based neuronal activity level was assessed. Control mice consisted in animal kept food/water fasted.

L-NG viral transduction and pharmacogenetic modulation of ascending vagal activity. Prior to surgery, mice received an i.p. injection of buprenorphine (0.1mg/Kg, Buprecare,

Axience, Pantin, France) in saline (NaCl 0.9%), 30 minutes before anesthesia. Surgeries were performed under aseptic conditions and 1–2% isoflurane/oxygen anesthesia. Mice body temperature was maintained at 37°C using a heating pad. Surgical access to the left nodose/jugular complex was obtained by making an incision along the ventral surface of the neck and blunt dissection using bilateral surgical retractors placed in the neck region at the level of the nodose ganglia and to ensure its exposure and surgical access by applying lateral tension to the surrounding muscular/aorto-esophageal/blood vessels complex. Viral preparation consisted of AAV5-hSyn-DIO-hM4D(Gi)-mCherry (Addgene 44362-AAV-5, titer: 4.5 X 10^{12} vg/ml), AAV5-hSyn-DIO-hM3D(Gq)-mCherry (Addgene 44361-AAV5, titer: 3.5 X 10^{12} vg/ml), AAV5-hSyn-DIO-mCherry (Addgene 50459-AAV5, titer: 3.4 X 10^{13} vg/ml) and AAV1-hSyn-Cre (Addgene 105553-AAV1, titer: 2.6 X 10^{13} vg/ml). Viral mixtures were complemented with a 1/5 addition of AAV1-hSyn-Cre, and 0.05% Fast Green FCF (Sigma-Aldrich). An elongated glass capillary mounted on an Nanoject III microinjector (Drummond), filled with the corresponding viral mix, was inserted into the nodose/jugular complex, and 90 nl of the virus solution were injected per mouse, in three 30 nl injection bolus, at 6 nl/s. Success in injection of the nodose was determined by Fast Green Dye filling of the ganglia. For the L-NG pharmacogenetic inhibition or activation experiments (Fig. 3 and S2E), AAV5-hSyn-DIO-hM4D(Gi)-mCherry or AAV5-hSyn-DIO-hM3D(Gq)-mCherry, respectively, and AAV5-hSyn-DIO-mCherry as a control, was used. For the validation of the L-NG pharmacogenetic activatory and inhibitory approach (Fig. S2A-C), group1: AAV5-hSyn-DIO-mCherry only as a control, group 2: a 1/4 mixture of AAV5-hSyn-DIO-hM3D(Gq)-mCherry with AAV5-hSyn-DIO-mCherry, group 3: a 1/4 mixture of AAV5-hSyn-DIO-hM3D(Gq)-mCherry with AAV5-hSyn-DIO-hM4D(Gi)-mCherry, was used. Three weeks following the viral injection, pharmacogenetic ascending-vagal inhibition or activation was initiated by CNO (Enzo life sciences, Fisher scientific BML-NS105-0025) i.p. injections, at 2mg/kg in saline (NaCl 0.9%), 30 min before the start of the ISO-NOL and novelty exposure procedures.

Statistics. All experiments and data analyses were achieved blinded. Each experiment was replicated at least twice. Statistical analyses were performed with GraphPad Prism 8.0. No statistical methods were used to pre-determine sample size, or to randomize. Prior to further analysis, sample normality was tested using the D'Agostino and Pearson omnibus normality test. For statistical analysis, between two groups, unpaired t-test, with an assumption of equal variance was used and between three or more independent groups, ANOVA tests coupled to post hoc Sidak's multiple comparison tests was used. Fig. 1E statistical analysis, a two-way ANOVA with Fisher test was used. In behavioral experiments, outliers were identified using Grubbs' method ($\alpha=0.05$) and then removed. Figure legends indicate the number of subjects used in each experimental condition and the methods of statistical analysis. Data are expressed as mean ±SD. Statistical significance was set at $*P<0.05$, $**P < 0.01$, $***P < 0.001$, $****P < 0.0001$.

Funding. The project leading to this application has received funding from the European Union's Horizon 2020 research and innovation program under the Marie Sklodowska-Curie grant agreement No 708466 and from the Institut Pasteur Research Applications and Industrial Relations Department grant DARRI INNOV 43-2019.
